# First report of structural characteristics and polymorphisms of the prion protein gene in raccoon dogs: The possibility of prion disease-resistance

**DOI:** 10.3389/fvets.2022.989352

**Published:** 2022-09-20

**Authors:** Woo-Sung Jo, Yong-Chan Kim, Jae-Ku Oem, Byung-Hoon Jeong

**Affiliations:** ^1^Korea Zoonosis Research Institute, Jeonbuk National University, Iksan, South Korea; ^2^Department of Bioactive Material Sciences, Jeonbuk National University, Jeonju, South Korea; ^3^Laboratory of Veterinary Infectious Diseases, College of Veterinary Medicine, Jeonbuk National University, Iksan, South Korea

**Keywords:** prion protein gene, *PRNP*, prion diseases, raccoon dog, dog

## Abstract

Prion diseases are fatal degenerative encephalopathies caused by misfolded prion protein (PrP^Sc^) converted from normal prion protein (PrP^C^). Previous studies have reported that genetic polymorphisms of the prion protein gene (*PRNP*) play a critical role in susceptibility to prion diseases. In addition, prion disease-resistant animals showed unique structural features of prion protein (PrP) related to species-specific amino acids. However, investigations of genetic polymorphisms of the *PRNP* gene and structural characteristics of PrP have not been performed in raccoon dogs thus far. We investigated genetic polymorphisms of *PRNP* in 87 raccoon dogs using amplicon sequencing and analyzed the genotype, allele, haplotype frequencies, and linkage disequilibrium (LD) using Haploview version 4.2. In addition, we performed phylogenetic analysis and multiple sequence alignment (MSA) using MEGA X version 10.1.8 and Clustal X version 2.1, respectively. We estimated the impact of raccoon dog and Canidae family-specific amino acids using PolyPhen-2, PROVEAN, and AMYCO. Furthermore, we analyzed the effect of raccoon dog and Canidae family-specific amino acids using the AlphaFold2 and Swiss-PdbViewer programs. We found 4 novel single nucleotide polymorphisms (SNPs) of the raccoon dog *PRNP* gene. In addition, the raccoon dog PrP showed 99.61% identity and the closest genetic distance to dog PrP. Among the substitutions of Canidae-specific amino acids with interspecific amino acids, D163N showed increased amyloidogenic propensity, and R181H showed alterations of hydrogen bonds. Furthermore, electrostatic potentials were changed according to the substitutions of D163N and R181H. By comparing PrP between raccoon dogs and raccoons, R168K and K224R were found to be related to changes in hydrogen bonds, and K224R altered the electrostatic potential of raccoon dog PrP. In the present study, we first reported 4 novel synonymous SNPs of the raccoon dog *PRNP* gene. We also identified that the PrP of raccoon dog has high homology (99.61%) with PrP of dog, which is a prion-resistant animal. In addition, raccoon dog PrP-specific amino acids are related to low amyloid propensity and inherent characteristics of 3D structure of raccoon dog PrP compared to the PrP of prion-susceptible species.

## Introduction

Prion diseases are progressive neurodegenerative brain disorders induced by deleterious form of prion protein (PrP^Sc^) changed from benign form of prion protein (PrP^C^) (1). Since the outbreak of bovine spongiform encephalopathy (BSE) in the United Kingdom in the 1980s, prion diseases have been reported in a wide range of animals, including humans, cattle, deer, sheep, goats, cats, minks, and raccoons (2). According to earlier studies, genetic variations of the prion protein gene (*PRNP*) are significantly related to vulnerability to prion diseases (2). In humans, the M129V single nucleotide polymorphism (SNP) of *PRNP* is related to susceptibility to sporadic and variant Creutzfeldt-Jakob disease (CJD) (3). The M/M homozygote of this SNP is more frequently observed in sporadic and variant CJD. In cattle, 23-bp and 12-bp insertion/deletion polymorphisms located on the regulatory region of the bovine *PRNP* gene are associated with susceptibility to BSE (4). In sheep, the V/R/Q and A/R/R haplotypes at codons 136, 154, and 171 of ovine PrP confer susceptibility and resistance to classical scrapie, respectively (2, 4, 5). In addition, several studies have shown that variants at codons 142, 143, 146, 154, 171, 211, and 222 of the caprine *PRNP* gene affect scrapie progression in goats (2, 6, 7). Furthermore, *PRNP* SNPs are related to chronic wastiing disease (CWD) susceptibility in several deer species (moose: S100Q; Rocky Mountain elk: M132L; white-tailed deer: Q95H, G96S, A116G) (2, 8).

However, prion-infected cases were not reported in dogs during the BSE outbreak. In addition, dog PrP showed resistance to PrP^Sc^ conversion using several prion agents including BSE, scrapie, and CWD by protein misfolding cyclic amplification (PMCA) (9). *In silico* studies of dog PrP structure indicated that the D163 of dog PrP confers extraordinary stability of dog PrP *via* expanded helix 1 and a negative surface charge and is associated with resistance to prion diseases (10). Furthermore, transgenic mice carrying mouse PrP with the dog-specific amino acid, D158 showed resistance to intracerebral infection using several scrapie strains, including 301C, 22L, and Rocky Mountain Laboratory (RML) (10). Intriguingly, recent studies have reported that raccoons (*Procyon lotor*) can be infected with CWD by ingesting a CWD infected cervid carcass as a scavenger (11, 12).

Raccoon dog (*Nyctereutes procyonoides*) is an indigenous species that inhabits Korea, China, Japan, Vietnam, and East Europe. As indicated by the name, raccoon dogs have a similar appearance and habitats to both raccoons and dogs. However, the possibility of prion infection in raccoon dogs has not been investigated thus far. In addition, although polymorphisms of the *PRNP* gene have been reported in various hosts, *PRNP* polymorphisms have not been investigated in raccoon dogs (13–21).

In the present study, we investigated genetic polymorphisms of the *PRNP* gene of 87 raccoon dogs inhabiting the Republic of Korea. In addition, we performed multiple sequence alignment and phylogenetic analysis using PrP sequences of various species, including raccoon dogs. Furthermore, we annotated the effect of Canidae-specific amino acids on raccoon dog PrP using *in silico* programs, including PolyPhen-2, PROVEAN, AMYCO, AlphaFold2 and Swiss-PdbViewer.

## Materials and methods

### Sample statements

Tissue samples from 87 raccoon dogs were provided by the College of Veterinary Medicine at Jeonbuk National University and the National Institute of Environmental Research in the Republic of Korea.

### Genomic DNA extraction

Genomic DNA was extracted from 20 mg of peripheral tissue samples, including liver, lung, skin, muscle, and kidney of raccoon dogs using a Bead Genomic DNA Prep Kit (BioFACT, Daejeon, Korea).

### Genetic analysis

To amplify the raccoon dog *PRNP* gene, polymerase chain reaction (PCR) was performed using *Taq*^Basic^ DNA Polymerase Kit (BioFACT, Daejeon, Korea) with forward primer: 5'-GAGCACACGTAGGATGCTGA-3' and reverse primer: 5'-CCTCCCCCAACCTGTAAAA-3', which were designed based on the nucleotide sequences of the raccoon dog *PRNP* gene (GenBank accession number: EU341507.1). The PCR products (876 bp) contained the entire open reading frame (ORF) of the raccoon dog *PRNP* gene. The PCR reagent contained 2.5 μl of 10X *Taq* reaction buffer with 25 mM MgCl_2_, 5 μl of 5X Band Helper™, 0.5 μl of each 10 mM dNTP mixture, 10 pmol each primer, 0.25 μl of *Taq* DNA polymerase (5 U/μl), 1–2 μl of raccoon dog genomic DNA (50–200 ng/μl) and sterile deionized water up to 25 μl. The PCR conditions were as follows: predenaturation at 95 °C for 2 min, followed by 34 cycles of denaturation at 95 °C for 20 s, annealing at 57 °C for 40 s and elongation at 72 °C for 1 min and one cycle of 72 °C for 5 min. The PCR products were separated on a 1% agarose gel stained with ethidium bromide (EtBr). The separated PCR products were purified by a FavorPrep™ GEL/PCR Purification Mini Kit (FAVORGEN, Pingtung County, Taiwan) and directly sequenced using an ABI 3730 sequencer (ABI, Foster City, CA, USA). The sequencing results were read by Finch TV software (Geospiza Inc., Seattle, WA, USA).

### Statistical analysis

To determine whether polymorphisms of the raccoon dog *PRNP* gene were in Hardy-Weinberg equilibrium (HWE), the chi-squared test was performed using Haploview version 4.2 (Broad Institute, Cambridge, MA, USA). Haplotype and linkage disequilibrium (LD) analyses were performed using Haploview version 4.2. LD was represented as *r*^2^ values.

### Phylogenetic analysis

The amino acid sequences of PrP of 14 species including human, mouse, cattle, sika deer, red deer, elk, sheep, goat, horse, raccoon, mink, cat, dog, and raccoon dog were collected from GenBank at the National Center for Biotechnology Information (NCBI). Detailed information on the amino acid sequences of the PrPs is given in Supplementary Table 1. Phylogenetic analysis was performed by MEGA X version 10.1.8 (22). The evolutionary tree was constructed by the neighbor-joining method (5,000 bootstraps). The length of the tree branch representing the evolutionary distances was estimated using the Poisson correction method and is shown in units of the number of amino acid substitutions per site.

### Multiple sequence alignments of amino acid sequences of PrPs

Multiple sequence alignments of amino acid sequences of PrPs in 14 species including human, mouse, cattle, sika deer, red deer, elk, sheep, goat, horse, raccoon, mink, cat, dog, and raccoon dog were performed using ClustalX version 2.1 (Conway Institute, UCD Dublin, Dublin, Ireland).

### *In silico* analysis of the effect on raccoon dog PrP according to amino acid substitutions

To evaluate the effect on raccoon dog PrP according to amino acid substitution, PolyPhen-2 (http://genetics.bwh.harvard.edu/pph2/index.shtml), PROVEAN (http://provean.jcvi.org/seq_submit.php), and AMYCO (http://bioinf.uab.es/amycov04/) were used. PolyPhen-2 is a program that evaluates the effect of amino acid substitution according to the position-specific independent count score and represents three types: “probably damaging,” “possibly damaging” and “benign.” PROVEAN estimates the impact of protein substitution on protein function. PROVEAN gives a score calculated by clustering BLAST hits according to the homologs collected from the NCBI NR protein database. The PROVEAN score represents “deleterious” below −2.5 and “neutral” above −2.5. AMYCO evaluates the impact of protein substitution on the aggregation propensity of proteins. AMYCO uses the pWALTZ and PAPA algorithms to calculate the impact of amyloidogenic sequences, which is represented as the PSEP score. A score higher than 0.78 indicates a strong aggregation propensity, and a score lower than 0.45 indicates a weak aggregation propensity.

### 3D structure analysis of raccoon dog PrP

3D structure prediction of raccoon dog PrP was performed by AlphaFold2 based on machine learning. The confidence of predicted structure was evaluated by the predicted local distance difference test (pLDDT) value on a scale from 0 to 100. Hydrogen bond and electrostatic potential changes according to the amino acid substitutions were predicted by the Swiss-PdbViewer program (23). Hydrogen bonds were predicted according to the interatomic distance and amino acid types and estimated if hydrogen was in the range from 1.2 to 2.76 Å of a compatible donor atom. Electrostatic potential was calculated by the Poisson-Boltzmann equation using the surface charges of the amino acids exposed on the protein surface.

## Results

### Identification of novel polymorphisms of the raccoon dog *PRNP* gene

To amplify the raccoon dog *PRNP* gene, we performed PCR using *PRNP* gene specific primers. The nucleotide sequences of the PCR products were identical to those of the raccoon dog *PRNP* gene registered in GenBank (Gene ID: EU341507.1). A total of 87 samples of raccoon dogs were used for PCR to investigate polymorphisms of raccoon dog *PRNP*.

**Figure 1:**
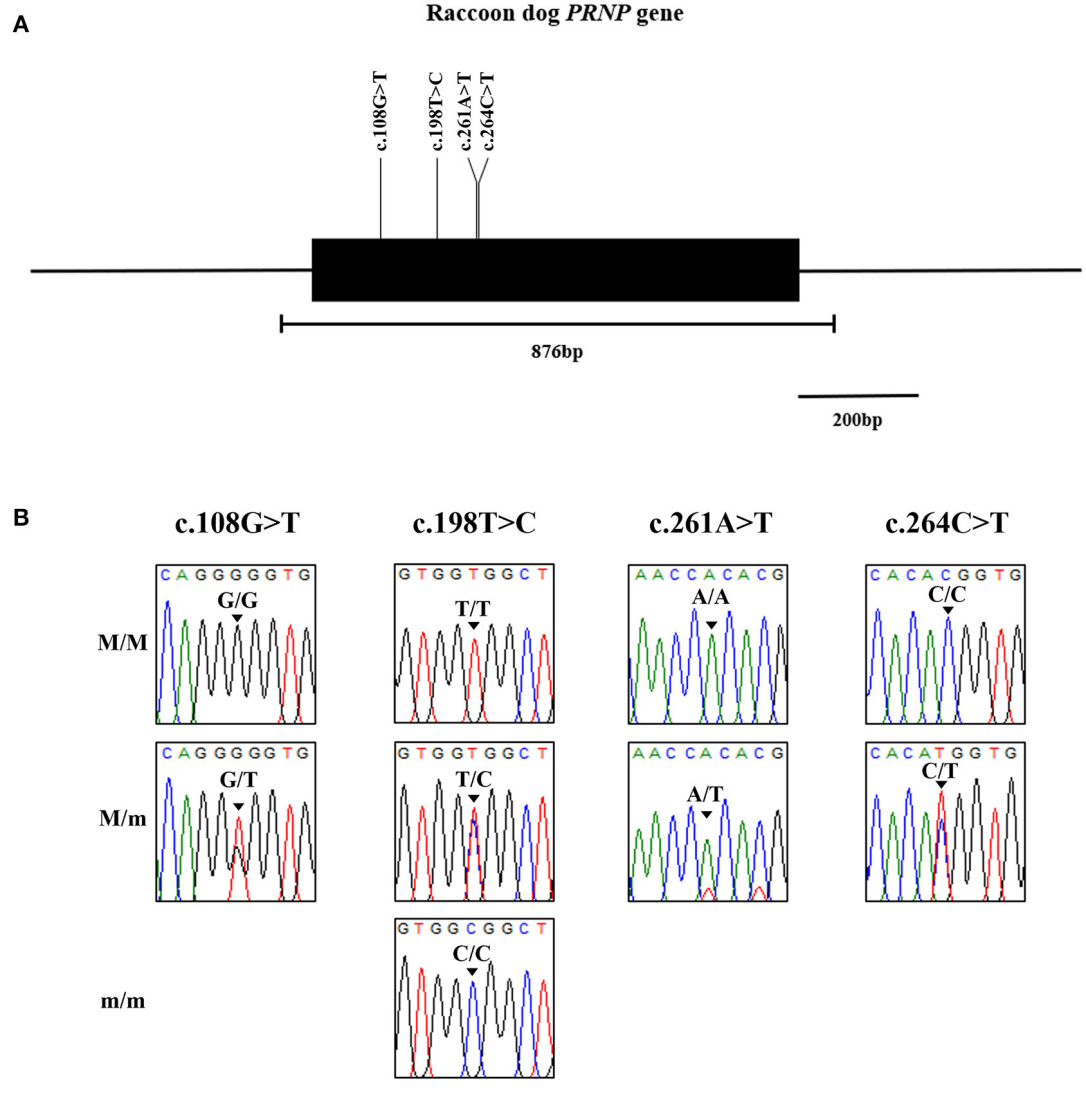
Identification of novel genetic polymorphisms of the prion protein gene (*PRNP*) in 87 raccoon dogs. **(A)** Simplified gene map of the raccoon dog *PRNP* gene. The open reading frame (ORF) is indicated by a shaded block (774 bp). The edged horizontal bar indicates the regions sequenced (876 bp). Vertical lines indicate the novel genetic polymorphisms identified in this study. **(B)** Electropherograms of 4 novel single nucleotide polymorphisms (SNPs) of the *PRNP* gene found in raccoon dogs. The colors of the peaks indicate each base of nucleotides (green: adenine; red: thymine; blue: cytosine; black: guanine). M/M: major homozygote; M/m: heterozygote; m/m: minor homozygote.

We identified 4 novel synonymous SNPs, c.108G > T, c.198T > C, c.261A > T and c.264C > T (Figure 1). Among 87 raccoon dogs, 75 (86.21%) were homozygous for G and 12 (13.79%) were heterozygous at c.108G > T. The allele frequency of c.108G > T was 162 G alleles (93.1%) and 12 T alleles (6.9%). At c.198T > C, 46 (52.87%) were homozygous for T, 4 (4.6%) were homozygous for C, and 37 (42.53%) were heterozygous. The allele frequency of c.198T > C was 129 T alleles (74.14%) and 45 C alleles (25.86%). At c.261A > T, 86 (98.85%) were homozygous for A and 1 (1.15%) was heterozygous. The allele frequency of c.261C>T was 173 G alleles (99.43%) and 1 T allele (0.57%). At c.264C > T, 85 (97.7%) were homozygous for C and 2 (2.3%) were heterozygous. The allele frequency of c.264C > T was 172 C alleles (98.85%) and 2 T alleles (1.15%). All polymorphisms found in this study were in HWE (Table 1). We investigated LD values among 4 polymorphisms of the raccoon dog *PRNP* gene (Table 2). Strong LD (*r*^2^ > 0.333) was observed between c.261A > T and c.264C > T (0.497). Next, we performed haplotype analysis among the 4 polymorphisms (Table 3). The most frequently observed haplotype was GTAC (74.1%), followed by GCAC (17.8%), TCAC (6.9%), GCAT (0.6%), and GCTT (0.6%).

**Table 1:** Genotype and allele frequencies of *PRNP* polymorphisms in raccoon dogs.

**Polymorphisms**	**Genotype frequency**, ***n*** **(%)**			**Allele frequency**, ***n*** **(%)**		HWE^*^
c.108G > T (G36G)	GG	GT	TT	G	T	0.490
	75 (86.21)	12 (13.79)	0 (0)	162 (93.1)	12 (6.9)	
c.198T > C (G66G)	TT	TC	CC	T	C	0.309
	46 (52.87)	37 (42.53)	4 (4.6)	129 (74.14)	45 (25.86)	
c.261A > T (P87P)	AA	AT	TT	A	T	0.957
	86 (98.85)	1 (1.15)	0 (0)	173 (99.43)	1 (0.57)	
c.264C > T (H88H)	CC	CT	TT	C	T	0.914
	85 (97.7)	2 (2.3)	0 (0)	172 (98.85)	2 (1.15)	

* Hardy-Weinberg equilibrium.

**Table 2:** Linkage disequilibrium (LD) analysis of 4 *PRNP* polymorphisms of raccoon dogs.

***r* ^2^**	**c.108G > T**	**c.198T > C**	**c.261A > T**	**c.264C > T**
c.108G > T	–	0.212	0	0.001
c.198T > C	–	–	0.017	0.033
c.261A > T	–	–	–	**0.497**
c.264C > T	–	–	–	–

Bold: strong LD (r^2^>0.333).

**Table 3:** Haplotype frequencies of 4 *PRNP* polymorphysms in raccoon dogs.

**Haplotypes**	**c.108G > T**	**c.198T > C**	**c.261A > T**	**c.264C > T**	**Frequency**
					**(*****n*** **=** **87)**
ht1	G	T	A	C	129 (0.741)
ht2	G	C	A	C	31 (0.178)
ht3	T	C	A	C	12 (0.069)
ht4	G	C	A	T	1 (0.006)
ht5	G	C	T	T	1 (0.006)

### Phylogenetic analysis and multiple sequence alignment of amino acid sequences of PrPs

**Figure 2:**
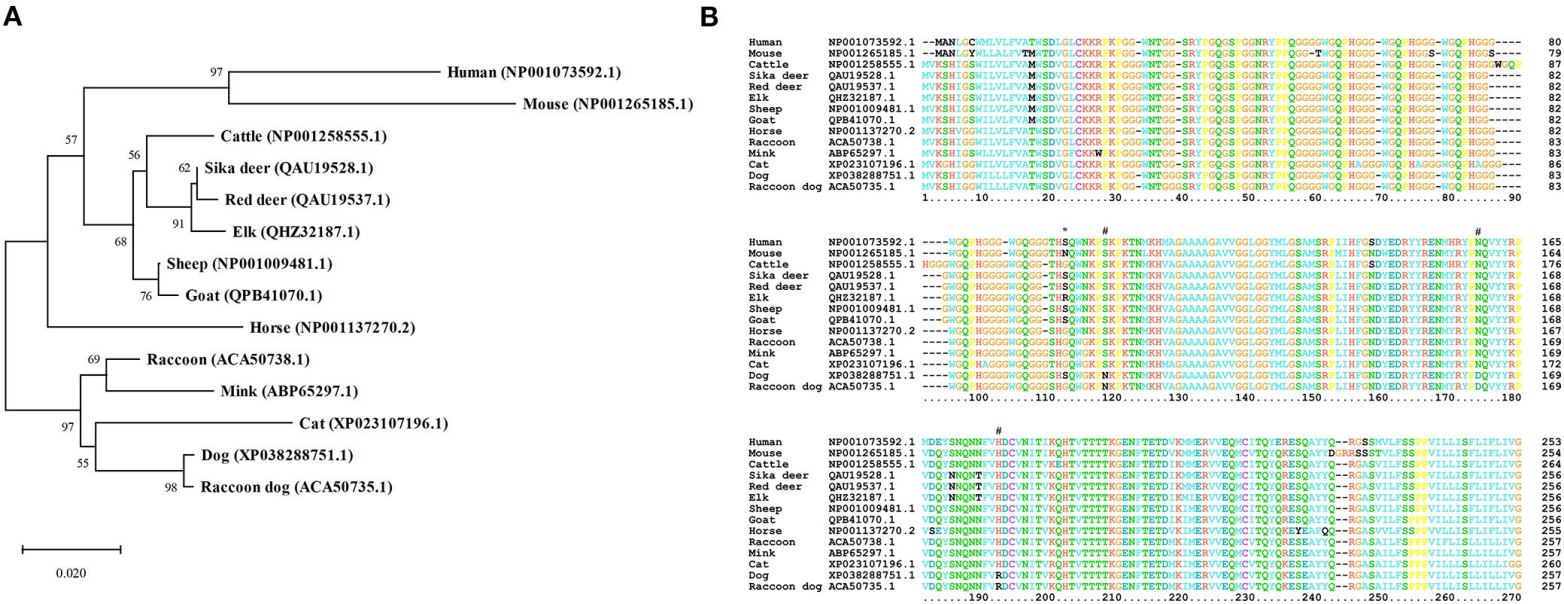
Phylogenetic tree and comparison of amino acid sequences of prion proteins (PrPs) in 14 species. **(A)** A phylogenetic tree was constructed by MEGA X using amino acid sequences of the PrPs from 14 species. Detailed information on the 14 species is described in Supplementary Table 1. The phylogenetic tree was drawn using neighbor-joining methods with 5,000 bootstraps. The length of the branches indicates the evolutionary distance. **(B)** Multiple sequence alignment was performed by ClustalX using PrP sequences of the 14 species described in Supplementary Table 1. The colors of the text indicate the chemical properties of amino acids (green: polar uncharged; blue: hydrophobic; cyan: negatively charged; red: positively charged; orange: glycine; yellow: proline; pink: cysteine; black: out of consensus). The asterisk indicates a raccoon dog-specific amino acid. Sharps indicate Canidae family-specific amino acids.

We constructed a phylogenetic tree to evaluate the evolutionary relationship between raccoon dog and prion disease-susceptible species (Figure 2A). In brief, raccoon dogs showed the closest genetic distance to dogs, known as prion disease-resistant animals. The horse, another prion disease-resistant animal, constituted an independent clade. In contrast, mice showed the farthest genetic distance from raccoon dogs. Sheep, goats, the Cervidae family and cattle, known as hosts of prion diseases, showed relatively greater genetic distances from raccoon dogs. Notably, raccoons showed a quite greater genetic distance from raccoon dogs. Instead, the genetically closest species to raccoons were minks and cats, known as prion disease-susceptible animals. The apparent close proximity of human and mouse PrP sequences in the phylogenetic tree is an artifact of the small number of species outside of the ungulate and Carnivora families.

To find raccoon dog-specific amino acids, we performed multiple sequence alignment of amino acid sequences of PrPs in 14 species including human, mouse, cattle, sika deer, red deer, elk, sheep, goat, horse, raccoon, mink, cat, dog, and raccoon dog using ClustalX version 2.1 (Figure 2B). We found only one raccoon dog specific amino acid, D163. Notably, raccoon dog PrP showed 99.61% identity with dog PrP. The Canidae family, including raccoon dogs and dogs shared three Canidae family-specific amino acids, including N107, D163, and R181.

### *In silico* evaluation of the effect of canidae family-specific amino acids on raccoon dog PrP

**Figure 3:**
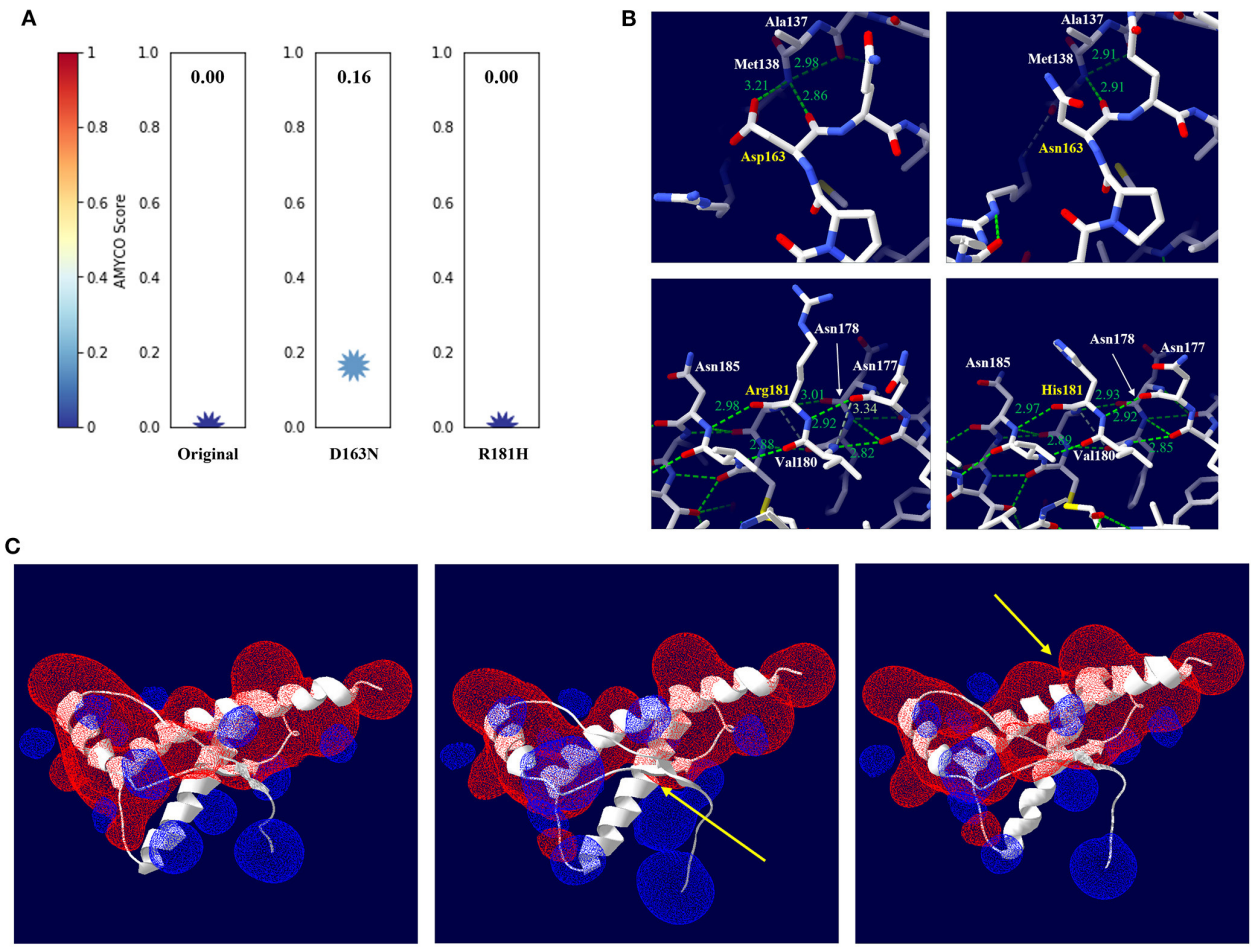
*In silico* evaluation of impact according to amino acid substitutions from Canidae family-specific amino acids to interspecies-conversed amino acids. **(A)** Evaluation of the impact according to the amino acid substitutions by AMYCO. **(B)** Hydrogen bond analysis according to substitutions by the Swiss-PdbViewer program. The upper left panel indicates raccoon dog PrP with the D163 allele. The upper right panel indicates raccoon dog PrP with the N163 allele. The lower left panel indicates raccoon dog PrP with the R181 allele. The lower right panel indicates raccoon dog PrP with the H181 allele. The green dotted lines indicate hydrogen bonds. The green numbers indicate the distance of the hydrogen bonds. **(C)** Electrostatic potential prediction according to substitutions by the Poisson-Boltzmann equation in the Swiss-PdbViewer program. The left panel indicates the electrostatic potential of wild-type raccoon dog PrP. The middle panel indicates the electrostatic potential of raccoon dog PrP with the N163 allele. The right panel indicates the electrostatic potential of raccoon dog PrP with the H181 allele. Positive potentials are noted in blue. Negative potentials are drawn in red. The yellow arrows highlight remarkable electrostatic potential changes compared with the wild-type raccoon dog PrP.

To evaluate the effect of Canidae family-specific amino acids on raccoon dog PrP, we substituted Canidae family-specific amino acids with interspecies conserved amino acids in prion disease-susceptible species and evaluated the effect of the substitutions on raccoon dog PrP using *in silico* tools. Notably, all substitutions, including N107S, D163N and R181H, were predicted to have no significant effect on the function and structure of raccoon dog PrP by PolyPhen-2 and PROVEAN (Supplementary Table 2). To evaluate the potential amyloidogenic propensity of the substitution, we carried out *in silico* analysis using AMYCO. The wild-type raccoon dog PrP was predicted to have a low amyloidogenic propensity with a score of 0. However, the raccoon dog PrP with the N163 allele (score 0.16) showed a higher amyloidogenic propensity than the wild-type PrP with D163. However, since the score was lower than 0.45, it indicated a weak aggregation propensity. The raccoon dog PrP with the H181 allele (score 0) showed an amyloidogenic propensity identical to that of the wild-type PrP with R181 (Figure 3A).

We evaluated the impact of the substitution on the 3D structure and hydrogen bonds of raccoon dog PrP by AlphaFold2 and Swiss-PdbViewer. According to plDDT value, the predicted raccoon dog structures, including the wild type of raccoon dog PrP and raccoon dog PrPs with N163 and H181 alleles showed high confidence (Supplementary Figures 1A–C). At codon 163, D163 was predicted to have 2 hydrogen bonds with M138 (2.86 Å, 3.21 Å) (Figure 3B, upper left). Notably, N163 was predicted to have one hydrogen bond with M138 (2.91 Å) (Figure 3B, upper right). At codon 181, R181 was predicted to have 3 hydrogen bonds with N177 (2.92 Å), N178 (3.01 Å), and N185 (2.98 Å) (Figure 3B, lower left). H181 was predicted to have 3 hydrogen bonds with N177 (2.92 Å), N178 (2.92 Å), and N185 (2.97 Å) (Figure 3B, lower right).

We evaluated the effect of the substitutions on raccoon dog PrP in electrostatic potential by Swiss-PdbViewer. Intriguingly, the electrostatic potential of raccoon dog PrP showed significant changes according to the substitution (Figure 3C). In raccoon dog PrP with N163, the negative charge located on codons 162–164 was smaller than that in wild-type PrP with D163. In addition, the positive charge located on codons 138–140 was expanded (Figure 3C, middle). In raccoon dog PrP with H181, the positive charge located on the adjacent region of codon 181 was smaller than that in wild-type PrP with R181. In addition, the negative charge located on the adjacent region of codon 176 was expanded (Figure 3C, right).

### Comparison of amino acid sequences between raccoon dog and raccoon PrPs

**Figure 4:**
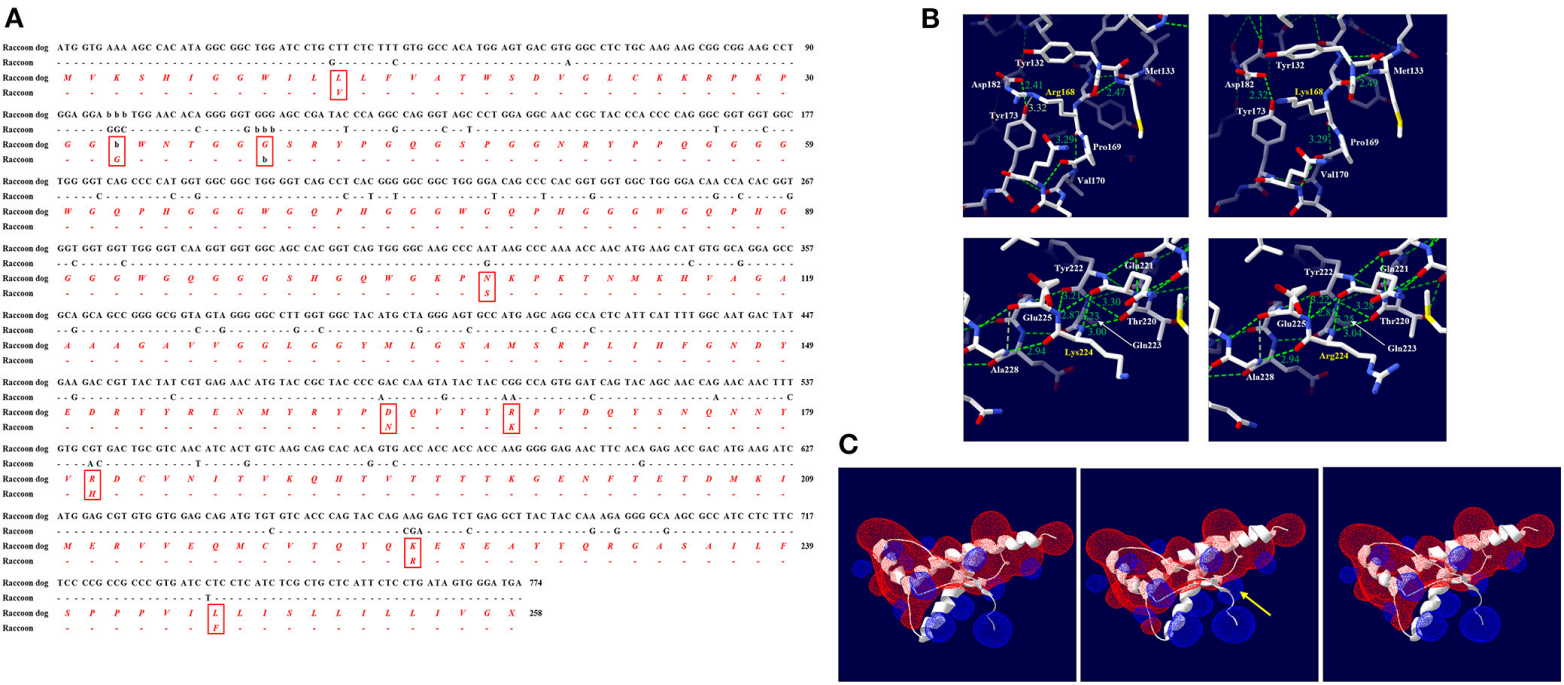
Comparison of the ORF of the *PRNP* gene between raccoon dogs and raccoons and *in silico* evaluation of the impact of raccoon-specific amino acid substitutions on raccoon dog PrP. **(A)** The black text indicates nucleotide sequences, and the red and italic texts indicate amino acid sequences. (-: identical nucleotide or amino acid to raccoon; b: nucleotide or amino acid deletion; X: stop codon) **(B)** Hydrogen bond analysis according to substitutions by the Swiss-PdbViewer program. The upper left panel indicates raccoon dog PrP with the R168 allele. The upper right panel indicates raccoon dog PrP with the K168 allele. The lower left panel indicates raccoon dog PrP with the K224 allele. The lower right panel indicates raccoon dog PrP with the R224 allele. The green dotted lines indicate hydrogen bonds. The green numbers indicate the distance of the hydrogen bonds. **(C)** Electrostatic potential prediction according to substitutions by the Poisson-Boltzmann equation in the Swiss-PdbViewer program. The left panel indicates the electrostatic potential of wild-type raccoon dog PrP. The middle panel indicates the electrostatic potential of raccoon dog PrP with the R168K substitution. The right panel indicates the electrostatic potential of raccoon dog PrP with the K224R substitution. Positive potentials are drawn in blue. Negative potentials are drawn in red. The yellow arrows highlight remarkable electrostatic potential changes compared with the wild-type raccoon dog PrP.

We compared the DNA and amino acid sequences of the ORF in the *PRNP* gene between raccoon dogs and raccoons (Figure 4A). Raccoon dog and raccoon showed 91.38% identity in their DNA sequences. In addition, we found 9 different amino acids at codons 12, 32–33 (ins), 38 (del), 107, 163, 168, 181, 224, and 246. Significant effects of substitutions of raccoon dog-specific amino acids with raccoon-specific amino acids on raccoon dog PrP were not observed by PolyPhen-2 and PROVEAN (Supplementary Table 3). However, R168K and K224R caused significant changes in the hydrogen bonds of raccoon dog PrP (Figure 4B). According to plDDT value, the predicted raccoon dog structures, including the wild type of raccoon dog PrP and raccoon dog PrPs with N163 and H181 alleles showed high confidence (Supplementary Figures 1A,D,E). At codon 168, R168 was predicted to have one hydrogen bond with Y173 (3.32 Å) and one hydrogen bond with V170 (3.29 Å) (Figure 4B, upper left). K168 was predicted to also have one hydrogen bond with V170 (3.29 Å) (Figure 4B, upper right). At codon 224, K224 was predicted to have two hydrogen bonds with T220 (3.00 Å) and Q221 (3.23 Å) (Figure 4B, lower left). However, R224 was predicted to also have two hydrogen bonds with T220 (3.04 Å) and Q221 (3.25 Å) (Figure 4B, lower right).

In raccoon dog PrP with K168, the positive charge located on codon 168 was expanded (Figure 4C, middle). In raccoon dog PrP with R224, a significant change was not observed compared with the wild-type PrP with K224 (Figure 4C, right).

## Discussion

Unlike other Carnivora species, including cats and minks, prion diseases have not been reported in dogs, which belong to Canidae, and dogs have been considered to have prion resistance. However, there is a question of whether prion resistance is a unique feature of dogs or is a characteristic of Canidae. Thus, we investigated the *PRNP* gene, which plays a pivotal role in susceptibility to prion diseases in another Canidae, the raccoon dog. We found that raccoon dog PrP showed 99.61% identity with dog PrP and had only 1 different amino acid at codon 101 compared to dog PrP. Previous studies have reported that dog PrP has the genetic polymorphism S101G. S101G has no significant effect on dog PrP (9, 15). Since raccoon dogs have amino acid sequences identical to those of dog PrP carrying G101, it is reasonable that raccoon dogs may have prion resistance. However, the raccoon dog *PRNP* polymorphisms were investigated in a relatively small sample size, and further investigation in a larger sample size is highly desirable.

In addition to codon 163, to identify genetic features regarding prion resistance in the Canidae family, we performed multiple sequence alignments and found that Canidae PrP has specific amino acids at codons 107, 163, and 181 (Figure 2B). To evaluate the effect of these Canidae-specific amino acids, we substituted the Canidae-specific amino acids with interspecific-conserved amino acids and analyzed the effect using *in silico* programs (Supplementary Table 2, Figure 3). Intriguingly, raccoon dog PrP carrying an interspecific-conserved amino acid at codon 163 was predicted to have a higher amyloidogenic propensity than wild-type raccoon dog PrP. These results were correlated with previous studies using PMCA and transgenic mice (9, 10). However, this substitution was not predicted to cause significant changes by PolyPhen-2 and PROVEAN, with no significant value. Since the two programs predict the effect of the substitution on protein based on the functional and structural changes, substitutions with amino acids that have similar structures and charges may not induce a significant effect on raccoon dog PrP. We also investigated the effect of amino acid substitutions on raccoon dog PrP using 3D structure analysis. Notably, prominent changes in hydrogen bonds were observed for alleles of D163N and R181H (Figure 3B, lower panel). In addition, electrostatic potential changes were also observed in raccoon dog PrP with the H181 allele or N163 allele compared to wild-type raccoon dog PrP (Figure 3C). Previous studies have reported that dipole moments induced by the electrostatic potential of PrP can drive PrP oligomerization. Since previous studies have suggested that electrostatic potential may affect prion aggregation, investigation of the effect of the alteration of electrostatic potential on prion aggregation according to specific amino acids is needed in the future (10, 24).

Previous studies have reported that cats are prion disease-susceptible animals, but dogs are prion disease-resistant animals, although both cats and dogs have similar habitats and prey (9, 25). The differences in susceptibility to prion diseases between cats and dogs are caused by differences in the amino acid sequences of each PrP (16). In particular, D163 of canine PrP, the dog-specific amino acid, plays a critical role in the stability of dog PrP by expanding helix 1 and affecting the surface charge, conferring resistance to various prion strains (10, 15). Similarly, raccoon dogs and raccoons have similar characteristics, including being major scavengers in the ecosystem, habitat, prey, and a similar appearance. Notably, although the raccoon dog also inhabits northern Europe, where the Cervidae family lives, the main host of CWD, prion diseases have not been reported in raccoon dogs thus far. Thus, we assumed that the differences in amino acid sequences of PrPs play a pivotal role in the difference in susceptibility to prion diseases between raccoon dogs and raccoons, as in the case of dogs and cats. In this study, we found 9 different amino acids between raccoon dogs and raccoons (Figure 4A). Notably, R168K and K224R were predicted to change the hydrogen bond and electrostatic potential, respectively (Figure 4B). Further study is needed to validate the effect of alterations of hydrogen bonds and electrostatic potential on prion aggregation in the future. However, since northern Europe has very low CWD incidence, careful interpretation of the phenomenon in which prion diseases have not been reported in raccoon dogs is needed. Further *in vivo* prion infection assay is needed to validate whether the raccoon dog is prion disease-resistant animal.

We found 4 novel synonymous SNPs of the raccoon dog *PRNP* gene in 87 raccoon dogs. We also found that the raccoon dog PrP has 99.61% identity with dog PrP and that raccoon dog PrP showed the closest genetic relationship with dog PrP. In addition, we found 3 Canidae family-specific amino acids. Among the substitutions of Canidae-specific amino acids with interspecific amino acids, D163N was predicted to elevate amyloidogenic propensity. Furthermore, D163N and R181H were associated with changes in the electrostatic potential of raccoon dog PrP. By comparison between raccoon dogs and raccoons, R168K and K224R were predicted to induce changes in the hydrogen bonds and electrostatic potential of raccoon dog PrP, respectively. To the best of our knowledge, this is the first study of genetic polymorphisms and structural features of the raccoon dog *PRNP* gene.

## Data availability statement

All data needed to evaluate the conclusions in the paper are present in the paper and/or the Supplementary materials. Additional supporting data are available from the corresponding authors upon reasonable request.

## Ethics statement

The animal study was reviewed and approved by Institute of Animal Care and Use Committee (IACUC) of Jeonbuk National University.

## Author contributions

W-SJ, Y-CK, and B-HJ analyzed the data, conceived, and designed the experiment. W-SJ performed the experiments. W-SJ, Y-CK, J-KO, and B-HJ wrote the article. All authors read and approved the final manuscript.

## Funding

This work was supported by a National Research Foundation of Korea (NRF) grant funded by the Korean government (MSIT) (2021R1A2C1013213 and 2022R1C1C2004792). This research was supported by the Basic Science Research Program through the National Research Foundation (NRF) of Korea funded by the Ministry of Education (2017R1A6A1A03015876 and 2021R1A6A3A010864).

## Conflict of interest

The authors declare that the research was conducted in the absence of any commercial or financial relationships that could be construed as a potential conflict of interest.

## Publisher's note

All claims expressed in this article are solely those of the authors and do not necessarily represent those of their affiliated organizations, or those of the publisher, the editors and the reviewers. Any product that may be evaluated in this article, or claim that may be made by its manufacturer, is not guaranteed or endorsed by the publisher.

## Supplementary material

Supplementary Figure 1 The tertiary structure of raccoon dog PrPs. **(A)** The tertiary structure of the wild-type raccoon dog PrP. **(B)** The tertiary structure of raccoon dog PrP with the N163 allele. **(C)** The tertiary structure of raccoon dog PrP with the H181 allele. **(D)** The tertiary structure of raccoon dog PrP with the K168 allele. **(E)** The tertiary structure of raccoon dog PrP with the R224 allele. pLDDT, predicted local distance difference test.
