# Supplementary figures and images for: First report of structural characteristics and polymorphisms of the prion protein gene in raccoon dogs: The possibility of prion disease-resistance
Source: Front Vet Sci. 2022 Sep 20;9:989352. doi: 10.3389/fvets.2022.989352 (PMC9530392; doi:10.3389/fvets.2022.989352)

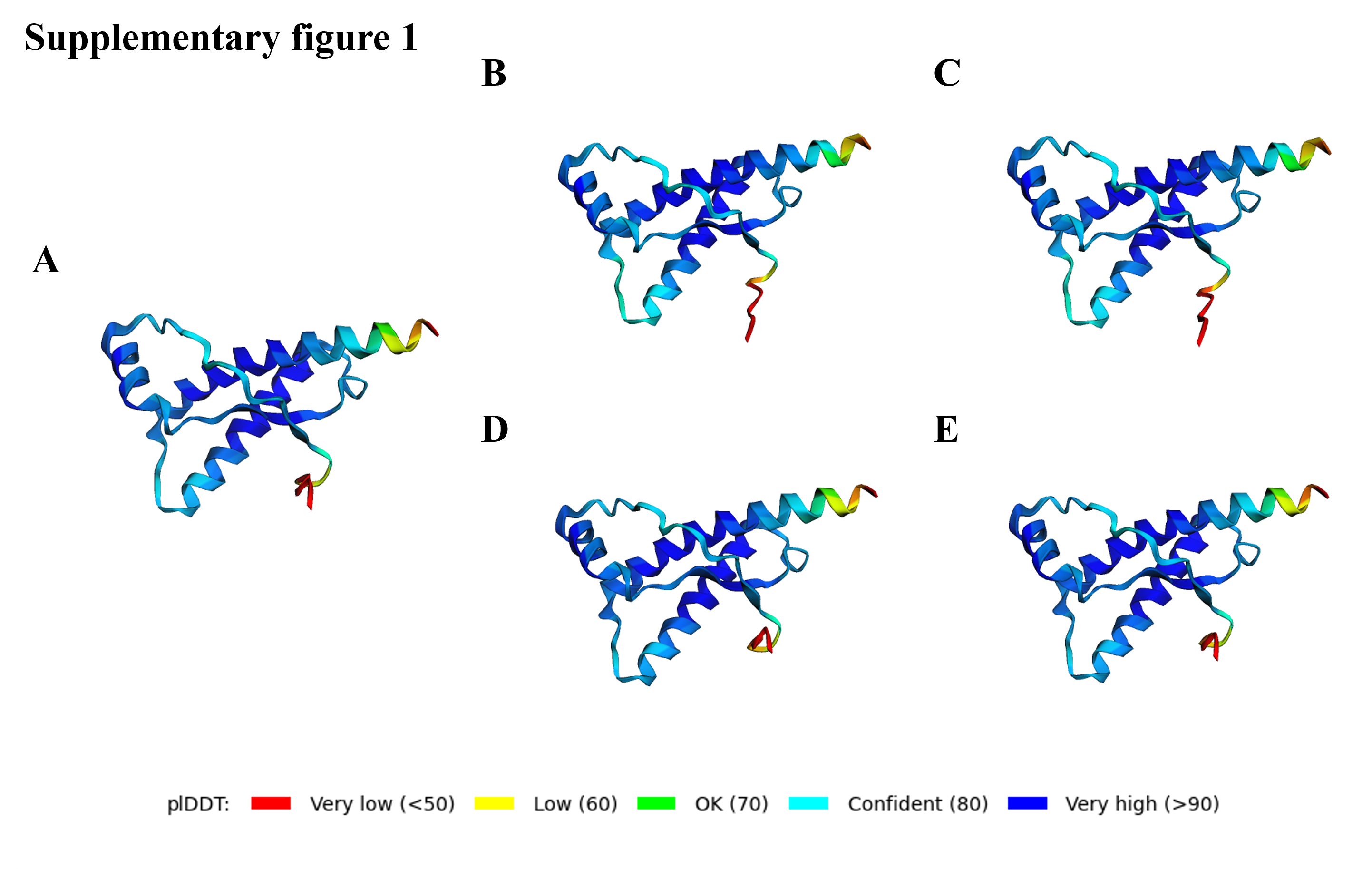

Supplement: Supplementary Figure 1 — The tertiary structure of raccoon dog PrPs. (A) The tertiary structure of the wild-type raccoon dog PrP. (B) The tertiary structure of raccoon dog PrP with the N163 allele. (C) The tertiary structure of raccoon dog PrP with the H181 allele. (D) The tertiary structure of raccoon dog PrP with the K168 allele. (E) The tertiary structure of raccoon dog PrP with the R224 allele. pLDDT, predicted local distance difference test. [file Image_1.tif]
