# Supplementary material for: First report of structural characteristics and polymorphisms of the prion protein gene in raccoon dogs: The possibility of prion disease-resistance
Source: Front Vet Sci. 2022 Sep 20;9:989352. doi: 10.3389/fvets.2022.989352 (PMC9530392; doi:10.3389/fvets.2022.989352)
Supplement: Supplementary file 2 [file Data_Sheet_1.docx]

**Supplementary Table 1. PrP sequence data of several species from the National Center for Biotechnology Information (NCBI).**

| Common name | Scientific name | Protein ID |
| --- | --- | --- |
| Human | *Homo sapiens* | NP001073592.1 |
| Mouse | *Mus musculus* | NP001265185.1 |
| Cattle | *Bos taurus* | NP001258555.1 |
| Sika deer | *Cervus nippon hortulorum* | QAU19528.1 |
| Red deer | *Cervus elaphus* | QAU19537.1 |
| Elk | *Alces alces alces* | QHZ32187.1 |
| Sheep | *Ovis aries* | NP001009481.1 |
| Goat | *Capra hircus* | QPB41070.1 |
| Horse | *Equus caballus* | NP001137270.2 |
| Raccoon | *Procyon lotor* | ACA50738.1 |
| Mink | *Neovison vison* | ABP65297.1 |
| Cat | *Felis catus* | XP023107196.1 |
| Dog | *Canis lupus familiaris* | XP038288751.1 |
| Raccoon dog | *Nyctereutes procyonoides* | ACA50735.1 |

**Supplementary Table 2. *In silico* analysis of raccoon dog PrP amino acid substitution to PrP of susceptible animals.**

|  | PolyPhen-2 (score) | PROVEAN (score) |
| --- | --- | --- |
| N107S | Benign (0.002) | Neutral (0.793) |
| D163N | Benign (0.000) | Neutral (0.689) |
| R181H | Benign (0.000) | Neutral (0.084) |

**Supplementary Table 3. *In silico* analysis of raccoon dog PrP amino acid substitution to raccoon PrP.**

|  | PolyPhen-2 (score) | PROVEAN (score) |
| --- | --- | --- |
| L12V | Benign (0.000) | Neutral (0.553) |
| G32_W33insG | N/A | Neutral (1.976) |
| G38del | N/A | Neutral (1.329) |
| R168K | Benign (0.009) | Neutral (-0.269) |
| K224R | Benign (0.000) | Neutral (-0.240) |
| L246F | N/A | Neutral (-0.796) |
